# Gastric perforation in an adult male following nasogastric intubation

**DOI:** 10.1308/003588412X13171221502347

**Published:** 2012-10

**Authors:** P Daliya, TJ White, KR Makhdoomi

**Affiliations:** Sherwood Forest Hospitals NHS Foundation Trust,UK

**Keywords:** Gastric perforation, Nasogastric intubation, Fibromuscular dysplasia

## Abstract

**Introduction:**

Spontaneous gastric perforation is a well known surgical emergency which carries significant mortality and morbidity. Well documented causes in adults include peptic ulcer disease, drugs such as non-steroidal and gastric malignancy. Iatrogenic causes still remain relatively rare. We report an interesting case of an acutely unwell young man who developed gastric perforation secondary to nasogastric intubation.

**Case Report:**

A 32 year old man initially treated for gastroenteritis underwent laparotomy for acute intra-abdominal bleeding. This was found to be secondary to a ruptured left hepatic artery aneurysm which was subsequently embolised. Patient had multiple laparotomies, a nasogastric tube inserted at the second laparotomy was later found to be the cause of gastric perforation. On further investigation the patient’s multiple aneurysms were histologically confirmed to be secondary to fibromuscular dysplasia (FMD).

**Conclusion:**

We present here a case of gastric perforation from a nasogastric tube in an adult male and discussed its relevance to the diagnosis of FMD. This case highlights the importance of having a high index of suspicion for this complication when managing patients with severe abdominal sepsis.

Spontaneous gastric perforation can be life threatening and carries significant morbidity and mortality. The mostcommon cause is peptic ulcer disease secondary to pharmacological agents such as non-steroidal anti-inflammatory drugs (NSAIDs) or steroids and, less commonly, neoplasia.^1^ Iatrogenic causes such as nasogastric (NG) and orogastric tube placement are extremely rare despite their frequent use in surgical and critically ill patients.^1,2^ We present the case of a previously fit and well adult male patient who developed gastric perforation following NG intubation.

## Case history

A 32-year-old man was admitted under the medical team with a 2-day history of diarrhoea, vomiting and generalised abdominal pain. On admission his haemoglobin was 12.9g/dl and urea 10.3mmol/l. A working diagnosis of gastroenteritis was made and he was rehydrated with intravenous fluids. Within hours of admission he became acutely unwell with increasing abdominal pain, distension and signs of shock. His haemoglobin was now 6.3g/dl and clinical examination demonstrated signs of intra-abdominal bleeding.

An emergency laparotomy revealed free blood in the abdomen and a ruptured aneurysm of the left hepatic artery. As it was not possible to control the bleeding by ligation, the abdomen was packed and the patient taken to the angiography suite, where the left hepatic artery was embolised. In addition to the left hepatic artery aneurysm, angiography also revealed splenic and renal artery aneurysms.

**Figure 1:**
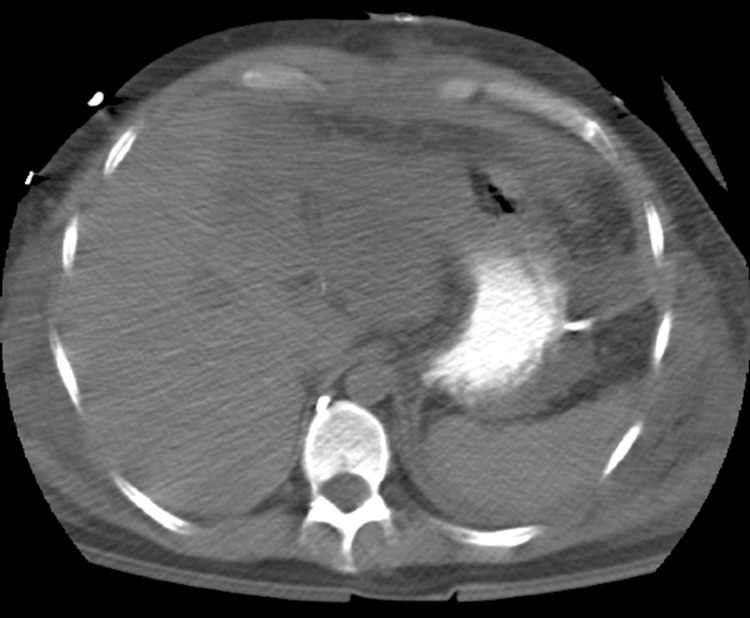
Nasogastric tube tip seen out with the stomach lumen instead of Nasogastric tube tip seen out with the stomach

**Figure 2:**
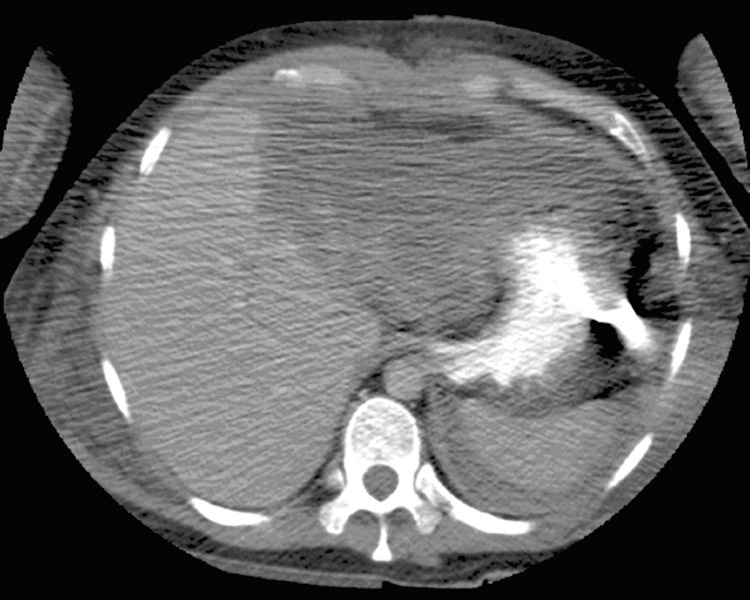
Extravasation of oral contrast from site of gastric perforation

**Figure 3:**
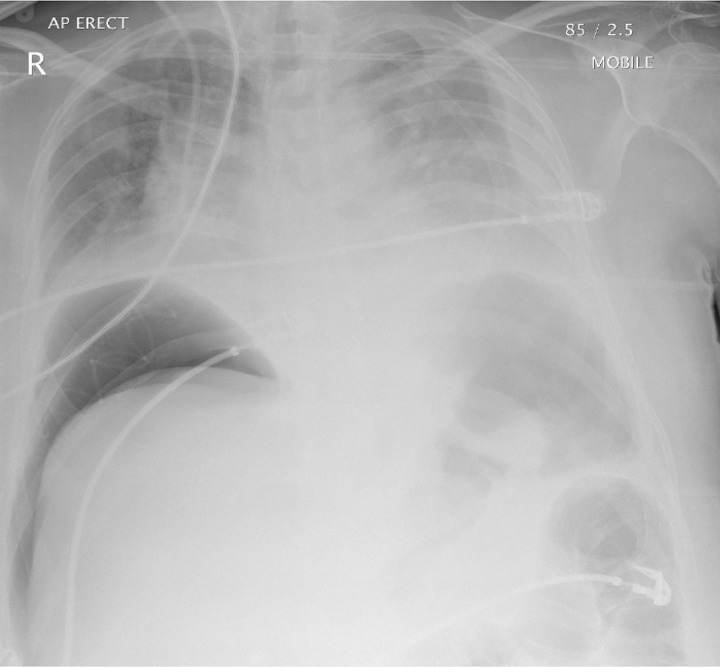
Erect chest x-ray confirming perforation following withdrawal of the nasogastric tube

A relaparotomy was performed 48 hours later to remove the abdominal pack and ligate the large splenic artery aneurysm. An NG tube was inserted during this operation, which also found the left lobe of the liver to be ischaemic but no gastric or intestinal compromise. One week later, the patient became acutely unwell, developed peritonitis and started to deteriorate. Abdominal computed tomography was arranged to visualise the liver necrosis after the embolisation and revealed the radiolucent tip of the NG tube outside the stomach wall (Figs 1 and 2) with extravasation of the oral contrast (Fig 2). The NG tube was pulled back and an erect chest x-ray demonstrated new free air under the diaphragms (Fig 3). An emergency relaparotomy confirmed gastric perforation, which was repaired by an omental patch.

Since discharge, histopathological analysis has confirmed the diagnosis of fibromuscular dysplasia (FMD) as the cause for this patient’s multiple aneurysms.

## Discussion

Oesophageal and pharyngo-oesophageal perforation are known complications of NG intubation in adults, unlike gastric perforation, which is more common in infants.^3^ A case series reported by Ghahremani *et al* in 1980 described six cases of gastric perforation following NG intubation.^1^ At least three of these patients had clearly documented risk factors, which could explain why perforation occurred so readily following NG tube placement (salicylate use, gastric anastomosis and metastatic gastro-oesophageal cancer).

In our case report, this previously well patient had no prior history of peptic ulcer disease or use of NSAIDs. We do not know for certain why an NG tube caused perforation in our patient as the stomach is thick-walled and well perfused in adults. Combined with the fact that the gastric blood supply was never compromised during embolisation, this renders the possibility of gastric ischaemia unlikely but not impossible.

A case reported by Lee *et al*^2^ and one by Ghahremani *et al*^1^ describe the occurrence of spontaneous gastric perforation following NG intubation. The action of gastrointestinal juices on the plastic NG tubing can cause previously flexible NG tubes to become rigid and potentially cause pressure ischaemia.^2,4^ Similar presentations have been described following NG intubation by a transoesophageal echocardiography probe^5^ and Linton tube.^1^

The only significant finding in our case was that of the diagnosis of FMD. FMD is a non-atherosclerotic, non-inflammatory arterial disease of unknown aetiology.^6,7^ It typically presents in the third to sixth decades with a 3:1 female-to-male preponderance. Muscular hyperplasia and fibrosis cause thickening of the arterial media with multifocal arterial stenosis. The renal artery is the most commonly affected, with the internal carotid artery being the next most common.^8,9^ Both of these were aneurysmal in our patient.

Radiologically, FMD is classified into three types (Table 1).^10^ According to this classification, our patient had the rare type III FMD, presenting as a ruptured left hepatic artery aneurysm.

**Table 1:** Fibromuscular dysplasia (FMD) classification

FMD type	Description
**I**	Affects 80% of cases consisting of a series of stenosis alternating with areas of dilatation. Histology shows medial fibroplasias.
**II**	Affects 15% of cases demonstrating unifocal with multifocal tubular stenosis. Histology shows intimal fibroplasias.
**III**	Affects 5% of cases involving a single wall of the artery but with resultant thinning that can cause true saccular aneurysm formation due to atypical FMD.

The aetiology of FMD is unknown although pathological studies demonstrate the involvement of the smooth muscle cells of the arterial media. Changes in the smooth muscle ultrastructure lead to fibrosis, post-stricture dilatation and subsequent saccular aneurysm formation.^9^ Little is known about the possible relations between FMD and other smooth muscle organs in the body. Visceral FMD has been reported in the gut, causing cases of mesenteric ischaemia^6^ and proctitis.^11^ However, there are no reports on its effect on the stomach.

In 2001 Vuong *et al* described a case of gastric ischaemia secondary to fibrodysplasia in von Recklinghausen’s disease^12^ while in 2003 Dinan *et al* reported a case of gastric wall weakening and perforation secondary to Duchenne muscular dystrophy.^13^ As we know, the stomach also consists of smooth muscle and it is a possibility that this could also be involved in the pathogenesis of FMD. Unfortunately, this can only be confirmed for certain by tissue sampling, which was not performed intra-operatively.

## Conclusions

We present an interesting case of gastric perforation in an adult caused by NG intubation on a background of FMD. Although extremely rare, one needs to be aware of this complication, particularly in patients with severe abdominal sepsis.

## References

[1] Ghahremani GG, Turner MA, Port RB. Iatrogenic intubation injuries of the upper gastrointestinal tract in adults. Gastrointest Radiol 1980; 5: 1–10 6153626 10.1007/BF01888590

[2] Lee SH, Kim MS, Kim KH*et al* Gastric perforation caused by nasogastric intubation in a patient on peritoneal dialysis. Korean J Nephrol 2007; 26: 250–253

[3] Gharehbaghy MM, Rafeey M. Acute gastric perforation in neonatal period. Med J Islamic Acad Sci 2001; 14: 67–69

[4] Ghahremani GG, Gould RJ. Nasogastric feeding tubes. Radiographic detection of complications. Dig Dis Sci 1965; 31: 574–585 3086062 10.1007/BF01318688

[5] Soong W, Afifi S, McGee EC. Delayed presentation of gastric perforation after transesophageal echocardiography for cardiac surgery. Anesthesiology 2006; 105: 1,273–1,274 17122591 10.1097/00000542-200612000-00028

[6] Rodriguez Urrego PA, Flanagan M, Tsai WS*et al* Massive gastrointestinal bleeding: an unusual case of asymptomatic extrarenal, visceral, fibromuscular dysplasia. World J Gastroenterol 2007; 13: 5,771–5,774 10.3748/wjg.v13.i43.5771 PMC4171267 17963307

[7] La Batide Alanore A, Perdu J, Ploufin PF. Fibromuscular dysplasia. Presse Med 2007; 36: 1,016–1,023 10.1016/j.lpm.2007.02.027 17442534

[8] Berceli SA. Hepatic and splenic artery aneurysms. Semin Vasc Surg 2005; 18: 196–201 16360576 10.1053/j.semvascsurg.2005.09.005

[9] Stanley JC, Wakefield TW. Arterial Fibrodysplasia. In: Rutherford RB, ed.Vascular Surgery. 5th ed. Philadelphia, PA: Saunders; 2000 pp387–408

[10] Fligelstone L. Chapter 9: Fibromuscular Dysplasia. In: Parvin SD, Earnshaw JJ, eds.Rare Vascular Disorders. Telford: TFM; 2005 pp53–57

[11] Quirke P, Campbell I, Talbot IC. Ischaemic proctitis and adventitial fibromuscular dysplasia of the superior rectal artery. Br J Surg 1984; 71: 33–38 6689966 10.1002/bjs.1800710110

[12] Vuong PN, Le Bourgeois P, Houissa-Vuong S*et al* Intimal muscular fibrodysplasia responsible for an ischemic gastric ulcer in a patient with a von Recklinghausen’s disease: a case report. J Mal Vasc 2001; 26: 65–68 11240532

[13] Dinan D, Levine MS, Gordon AR*et al* Gastric wall weakening resulting in separate perforations in a patient with Duchenne’s muscular dystrophy. Am J Roentgenol 2003; 181: 807–808 12933486 10.2214/ajr.181.3.1810807

